# CD56 expression predicts response to Daratumumab-based regimens

**DOI:** 10.1038/s41408-024-01051-5

**Published:** 2024-04-12

**Authors:** Allen J. Robinette, Laila Huric, Kameron Dona, Don Benson, Francesca Cottini

**Affiliations:** The Ohio State University, Columbus, OH USA

**Keywords:** Cancer therapy, Translational research

Dear Editor,

Daratumumab (Dara) and Isatuximab (Isa) are monoclonal antibodies that target CD38, a glycoprotein highly expressed by plasma cells and Multiple Myeloma (MM) cells [1]. While CD38 expression can predict response to anti-CD38 antibodies [1, 2] and strategies increasing CD38 expression improve responses to Dara [3, 4], other factors likely are relevant in the response to these drugs. CD56 or NCAM1 is a surface glycoprotein aberrantly expressed in more than seventy percent of patients with MM [5]. We recently showed that CD56 activates RSK2 and CREB1, and modulates expression of MCL1 and CRBN [6]. In this report, we investigate whether CD56 expression affects responses to anti-CD38 monoclonal antibodies, especially in combination with immunomodulatory drugs (IMiDs).

We first evaluated whether Dara or Isa changed CD56 levels. We observed downregulation of CD56 surface expression with Dara and Isa, with Dara being more effective than Isa (Fig. 1A and Fig. S1A). CD56 surface downregulation occurred at low concentration (Fig. S1B) and after just one hour of treatment (Fig. S1C). Since the downregulation was very quick, we hypothesize that CD56 could be internalized. We confirmed increased intracellular CD56 mainly with Dara (Fig. 1B and Fig. S1D–E). Treatment with Dara not only reduced the total protein levels of CD56 but also its downstream targets, MCL1 and BCL2, at protein (Fig. 1C) and mRNA levels (Fig. 1D), without affecting CD56 mRNA levels. Isa effects were less evident (Fig. S1F). As expected, Dara also reduced MCL1 levels in U266 cells overexpressing CD56 (Fig. S1G,H). These data suggest that Dara functions by triggering CD56 internalization and hence impeding its signaling, while Isa does not affect this pathway.

**Fig. 1:**
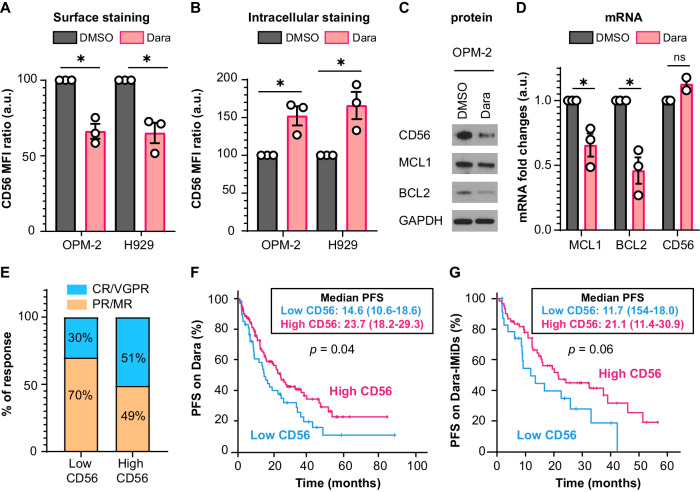
CD56 expression predicts response to daratumumab therapy. **A** Surface expression of CD56 in OPM-2 and H929 cells treated with DMSO or Dara 1 μg/mL for 48 h. Mean fluorescence intensity (MFI) value ratios are obtained normalizing to DMSO controls. n = 3 replicates. OPM-2 *p* = 0.041 (*); H929 *p* = 0.0358 (*). **B** Intracellular staining of CD56 in OPM-2 and H929 cells treated with DMSO or Dara 1 μg/mL for 1 h. MFI value ratios are obtained normalizing to DMSO controls. *n* = 3 replicates. OPM-2 *p* = 0.0145 (*); H929 *p* = 0.0217 (*). **C** Western blot analysis for CD56, MCL1, BCL2, and GAPDH in OPM-2 cells treated with DMSO and Dara 1 μg/mL for 48 h. **D** Quantitative PCR analysis for MCL1 (*p* = 0.05, *), BCL2 (*p* = 0.02, *), and CD56 (*p* = 0.12, ns) in OPM-2 cells treated with DMSO and Dara 1 μg/mL for 48 h (*n* = 2, 3 replicates). **E** Best responses to Dara therapy in 48 patients with <10% of CD56-expressing MM clonal cells (“Low CD56” group) or 104 patients with >10% of CD56-expressing MM clonal cells (“High CD56” group). In blue are reported the percentages of complete responses/very good partial responses (CR/VGPR), while in orange are the percentages of partial responses/minimal responses (PR/MR). *p* = 0.012. Percentages are included in the graph. **F** Progression-free survival (PFS) from the first day of Dara therapy in patients with less (*n* = 48, Low CD56-blue) or more than 10% of CD56-expressing MM clonal cells (*n* = 104, High CD56-fuchsia). Median PFS and 95% confidence intervals (CI) are reported in the insert of the plot. Log-rank *p* = 0.04. **G** PFS from the first day of Dara-IMiD therapy in patients with less (*n* = 24, Low CD56-blue) or more than 10% of CD56-expressing MM clonal cells (*n* = 60, High CD56-fuchsia). Immunomodulatory drugs (IMiDs) include lenalidomide or pomalidomide. Median PFS and 95% CI are reported in the insert of the plot. Log-rank *p* = 0.06.

To confirm whether CD56 expression could predict responses to Dara, we evaluated 152 patients (Table S1) who were treated with Dara single agent (*n* = 30), Dara in combination with IMiDs (lenalidomide or pomalidomide, *n* = 84), or Dara in combination with proteasome inhibitors (PIs- bortezomib or carfilzomib) (*n* = 38). CD38 was usually expressed by the primary clone (Median CD38 clone size = 97.25%), while CD56 clone size varied (Fig. S2A; median CD56 clone size = 42.90%). Low (*n* = 48) or High CD56 (*n* = 104) classification was based on a 10% cutoff of CD56-expressing clonal MM cells, as previously reported [6]. The two groups were well-balanced in terms of patient and disease characteristics (Table S1). Including all the patients treated with Dara independently of the combination regimen, we observed better responses to Dara in “High CD56” patients (CR/VGPR versus PR/MR, *p* = 0.012; Fig. 1E) and longer progression-free survival (PFS) from first day of Dara therapy (HR = 0.65, 95% CI: 0.43–0.98, *p* = 0.04; Fig. 1F), with a median PFS of 23.7 versus 14.6 months. Adjusting for stage, race, cytogenetics (t(4;14), del(13q), t(11;14), del(17p), and 1q+ ), and CD38 clone size, PFS remained significant (HR = 0.45, 95% CI: 0.28–0.72, *p* < 0.001). Overall survival (OS) from the first day of Dara was not different between the two groups (HR = 1.36, 95% CI: 0.737–2.5, *p* = 0.322; Fig. S2B).

CD56 signaling protects the cells from the activity of lenalidomide by decreasing CRBN expression [6]. Therefore, we hypothesized that Dara-IMiDs combination could be particularly effective in the “High CD56” group (Table S2). PFS was indeed superior in “High CD56” patients (HR = 0.58, 95% CI: 0.32–1.05, *p* = 0.06; Fig. 1G), with a median PFS of 21.1 versus 11.7 months. Adjusting for the same variables as above, PFS became even more significant (HR = 0.260, CI 95%: 0.12–0.55, *p* < 0.001). Conversely, the PFS effect was not statistically significant in patients treated with Dara-PIs (HR = 0.61, CI 95%: 0.23–1.67, *p* = 0.690; Fig. S2C) or Dara single agent (HR = 0.86, CI 95%: 0.402–1.827, *p* = 0.334; Fig. S2D). Thus, our data establish CD56 expression as a predictive marker of response to Dara in combination with IMiDs.

Since CD38 expression is linked to Dara response [1, 2], we incorporated median CD38 clone size into our prognostication model (Table S3), showing that patients in the “High CD38, Low CD56” group have the shortest median PFS of 8.7 months (All patients: Log-rank *p* < 0.001, Fig. 2A and Dara-IMiD patients: Log-rank *p* = 0.002, Fig. S3A). Interestingly, CD56 and CD38 are often co-expressed on MM cells, as noted by a positive correlation between CD38 and CD56 clone sizes in the 152 studied patients (*R* = 0.14, *p* < 0.0001, Fig. 2B), and between CD38 and CD56 mRNA levels in the CoMMpass dataset (*p* < 0.0001, Fig. S3B). We then analyzed whether CD56 could regulate CD38 expression. Overexpression of CD56 in U266 cells (Fig. S3C, D), which are negative for both CD56 and CD38 expression, and in MM.1S cells (Fig. S3E), which have low CD56 expression but high CD38 expression, significantly increased CD38 surface expression and CD38 mRNA levels (Fig. 2C and Fig. S3F). While we previously demonstrated that CD56 signals by CREB1 [6], CREB1 overexpression did not increase CD38 levels (Fig. S4A, B).

**Fig. 2:**
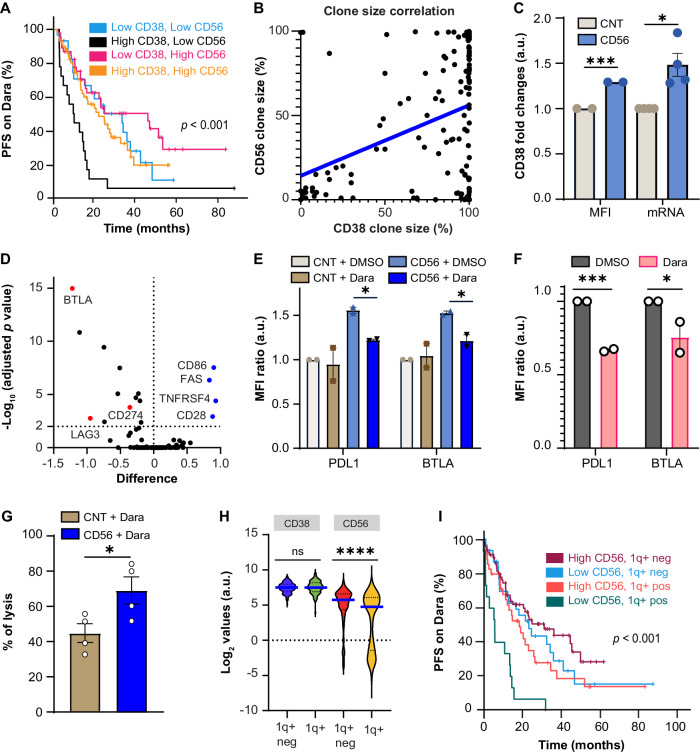
CD56, CD38, and 1q+ status regulate responses to anti-CD38 monoclonal antibodies. **A** Progression-free survival (PFS) from the first day of Dara therapy in patients with Low CD38, Low CD56 (*n* = 30, blue), High CD38, Low CD56 (*n* = 18, black), Low CD38, High CD56 (*n* = 46, fuchsia), and High CD38, High CD56 (*n* = 58, orange) disease. Median PFS and 95% confidence interval (CI) are reported in Table S3. Log-rank *p* < 0.001. **B** Regression analysis to correlate clone sizes of CD38- and CD56-expressing MM cells in 152 patients from our MM registry. Flow data were obtained before starting Dara therapy. *R* = 0.14; *p* < 0.0001. **C** Fold-changes of CD38 Mean Fluorescence Intensity (MFI) and mRNA levels in U266 control cells (CNT) or U266 cells overexpressing CD56. Ratio is normalized to the control cells. *n* = 2, 4 replicates. MFI *p* = 0.0009 (***); mRNA *p* = 0.032 (*). **D** Volcano plot showing differences in the RNA expression of 88 genes included in the described immune signature [8]. In red are highlighted upregulated genes, while in blue downregulated genes. **E** Fold-changes of PDL1 and BTLA MFI values in U266 control cells (CNT) or U266 cells overexpressing CD56 treated with DMSO or Dara 1 μg/mL for 48 h. Each MFI is normalized to the MFI of U266 control cells treated with DMSO. *n* = 2 replicates. CD56 + DMSO versus CD56 + Dara: CD274 *p* = 0.0112 (*); BTLA *p* = 0.0462 (*). **F** Fold-changes of PDL1 and BTLA MFI values in OPM-2 cells treated with DMSO or Dara 1 μg/mL for 48 h. n = 2 replicates. CD274 *p* = 0.0007 (***); BTLA *p* = 0.044 (*). **G** Percentage of 4-h Dara-induced ADCC lysis against MM.1S control cells (CNT) or MM.1S overexpressing CD56. Peripheral blood mononuclear cells derived from 4 healthy donors were used as effector cells, at an effector-to-target ratio of 25:1. *n* = 2 replicates. *p* = 0.04 (*). **H** CD38 and CD56 log_2_ expression values in 131 patients with 1q+ (either gains or amplifications) or 196 patients with normal 1q copy number and no other chromosomal abnormalities. CD38 1q+ versus 1q+ neg: *p* = 0.518, ns; CD56 1q+ versus 1q+ neg: *p* < 0.0001 (****). Blue solid lines indicate median values. Dotted black lines indicate the 25^th^ and 75^th^ percentiles. **I** PFS from the first day of Dara therapy in patients with Low CD56, 1q+ neg (*n* = 33, blue), Low CD56, 1q+ (*n* = 15, dark green), High CD56, 1q+ neg (*n* = 69, purple), and High CD56, 1q+ (*n* = 35, orange) disease. Median PFS and 95% CI are reported in Table S6. Log-rank *p* < 0.001.

The relationship between CD56 signaling and the immune system is still vastly unexplored in MM [7]. We first evaluated an immune signature ([8] and Table S4), which includes pro-inflammatory cytokines or molecules (e.g. IL2, IL6, CD3) and inhibitory markers (CD274, also known as PDL1, B and T lymphocyte attenuator-BTLA, LAG3, and TIM3). Gene Set Pathway analysis identified a correlation between CD56 expression and the above immune signature (Fig. S5A), with higher levels of inhibitory immune markers (Fig. 2D), including CD274 (*p* = 0.000025), LAG3 (*p* = 0.001732), and BTLA (*p* < 0.000001) and lower levels of FAS (*p* < 0.000001), CD28 (*p* < 0.0001), and CD86 (*p* < 0.000001) in “High CD56” patients. We then confirmed increase of surface expression of CD274 and BTLA with CD56 overexpression (Fig. S5B, C); while Dara reduced both markers in U266 cells overexpressing CD56 (Fig. 2E) and OPM-2 treated cells (Fig. 2F). Finally, Dara-induced antibody-dependent cellular cytotoxicity (ADCC) was greater in MM.1S overexpressing CD56 compared with control cells (Fig. 2G). Our findings convey that CD56 drives an immune inhibitory signature which can be reverted by Dara.

Isa did not modulate CD56 signaling; therefore, unsurprisingly no differences in PFS were noted based on CD56 clone size (HR = 1.530, CI 95%: 0.662–3.536, *p* = 0.31; Fig. S6A) in 32 patients (Table S5) treated with Isa-pomalidomide (*n* = 24, 75%) or Isa-carfilzomib (*n* = 8, 25%). While the number of prior lines clearly affected the overall PFS in our cohorts (Dara median prior lines = 3; Isa median prior lines = 8), consistently with previous literature [9], Isa still induced longer PFS in patients with amp(1q) (PFS: gain(1q) = 7.1 months; amp(1q) = Not-reached (NR), 1q+ negative = 14.3 months, *p* = 0.06; Fig. S6B), while Dara did not (PFS: gain(1q) = 15.6 months; amp(1q) = 10.8 months, 1q+ negative = 25.4 months, *p* < 0.001; Fig. S6C). Interestingly, median CD56 expression inversely correlates with 1q+ in the MMRF database (Fig. 2H), while the median CD38 clone size in patients with 1q+ was bigger (median = 98.3%) compared with patients without 1q+ (median = 88.2%) (*p* = 0.01, Fig. S6D). Including 1q+ status to subcategorize Low/High CD56 patients treated with Dara or Dara-IMiDs (Table S6, Fig. 2I and S6E), the combination of “Low CD56” and 1q+ had the worst outcomes (PFS: 6.7 or 3.9 months); “High CD56” status partially overcame 1q+ prognostic impact (PFS: 19.2 or 15.3 months) and “High CD56, 1q+ neg” patients had the best outcomes (PFS: 31.9 or 36.8 months). Overall, these data indicate opportunities to predict outcomes based on MM markers.

In summary, CD56 expression is a novel positive predictive factor of response to Dara-IMiD regimens but not Isa-based regimens, with Dara reducing CD56 surface expression and signaling. CD56 and CD38 are often co-expressed and CD56 regulates CD38 expression by a mechanism that is CREB1-independent. Moreover, patients with “High CD56” disease have a specific inhibitory immune signature, which can be reverted by Dara leading to increased ADCC, in agreement with previous data showing CD56 homophilic interaction between MM cells and NK cells [7].

1q+ status is associated with shorter PFS to Dara but not Isa, with CD56 expression only partially mitigating the negative prognosis impact of 1q+ status. These data are intriguingly and still not fully explained. Several genes are in the 1q21 cytoband, including MCL1 [10–12]. We hypothesize that CD56-mediated MCL1 downregulation is important for Dara-responses in patients without 1q+, while patients with 1q+ do not need CD56 to induce MCL1, and their increased responses to Isa are hence driven by other unidentified factors, such as alternative genes in the 1q21 cytoband or differences in immunity. This topic is the object of future ongoing research.

As a single-center study, our work has some limitations, including the low number of patients treated with Isa and the fact that the CD38-CD56 clone size determination preceded anti-CD38 monoclonal antibody therapy but was not assessed on the first day of therapy. Despite these limitations, our study paves the way for precision medicine in MM and raises important points to better understand the differences between the two anti-CD38 monoclonal antibodies. In conclusion, our data are clinically relevant, providing a novel potential strategy to select patients for the appropriate anti-CD38 therapy based on routine MM markers, such as 1q status and CD56 expression.

### Supplementary information

Supplementary Materials

## Data Availability

The datasets generated during and/or analyzed during the current study are available from the corresponding author upon reasonable request.
