# Supplementary material for: CD56 expression predicts response to Daratumumab-based regimens
Source: Blood Cancer J. 2024 Apr 12;14(1):62. doi: 10.1038/s41408-024-01051-5 (PMC11014999; doi:10.1038/s41408-024-01051-5)
Supplement: Supplementary file 1 — Supplementary Materials [file 41408_2024_1051_MOESM1_ESM.pdf]

Title: CD56 expression predicts response to Daratumumab-based regimens.

Authors:

Allen J. Robinette, Laila Huric, Kameron Dona, Don Benson, Francesca Cottini

[1] The Ohio State University, Columbus, OH, USA

Correspondence:

Francesca Cottini, MD (lead contact)

Assistant Professor of Medicine

Division of Hematology

The Ohio State University, College of Medicine

385G Wiseman Hall | 400 W 12th Ave |

Columbus OH 43210-1240 USA

Email: Francesca.cottini@osumc.edu

Competing interests: The authors declare no conflicts of interest.

Supplementary Contents:

1. Experimental Procedures:

- Cell lines, reagents, Flow cytometry analysis, western blot analysis, RNA extraction and quantitative real-time PCR analysis, and transfections.
- Immunophenotype for CD56 and CD38 clone size evaluation.
- Patient characteristics, definition of outcomes, and statistical analysis.
- RNA-sequencing Analysis.

2. Supplementary Tables:

- **Supplementary Table S1.** Characteristics of n = 152 patients treated with Daratumumab.
- **Supplementary Table S2.** Characteristics of n = 84 patients treated with Daratumumab in combination with immunomodulatory drugs (IMiDs).
- **Supplementary Table S3.** Median PFS based on CD38, and CD56 clone size subgrouping.
- **Supplementary Table S4.** Immune signature genes related to **Fig. 2D** and **Fig. S5A**.
- **Supplementary Table S5.** Characteristics of n = 32 patients treated with Isatuximab.
- **Supplementary Table S6.** Median PFS based on CD56 clone size, and 1q+ status subgrouping.

3. Supplementary Figures:

- **Supplementary Figure S1.** Modulation of CD56 and its downstream targets by anti-CD38 monoclonal antibodies.
- **Supplementary Figure S2.** Outcomes of patients treated with Dara based on CD56 clone size.
- **Supplementary Figure S3.** Role of CD38 and CD56 correlation in the response to Dara.
- **Supplementary Figure S4**. CREB1 does not regulate CD38 expression in MM.
- **Supplementary Figure S5.** CD56 regulates immune markers in MM.
- **Supplementary Figure S6.** Role of CD56 and 1q+ status in the response to anti-CD38 monoclonal antibodies.

1. Experimental Procedures:

Cell lines and culture: The Multiple Myeloma (MM) human cell lines H929, and MM.1S cells were purchased from American Type Culture Collection (ATCC), while the human MM cell lines U266 and OPM-2 cells were purchased from the Leibniz Institute DSMZ-German Collection of Microorganisms and Cell Cultures (DSMZ). All MM cell lines were cultured in RPMI-1640 medium containing 10% FBS (GIBCO, Life Technologies, Carlsbad, CA, United States), 2 μM/L glutamine, 10,000 U/mL penicillin G, and 10,000 μg/mL streptomycin (GIBCO, Life Technologies, Carlsbad, CA, United States) and maintained at 37 °C with 5% CO_2. Cells were used within 2-3 months after thawing. Cell lines were tested every 2-3 months for mycoplasma contamination using the MycoAlert mycoplasma detection kit (Lonza, Basel, Switzerland).

Reagents: Daratumumab (Cat. No. A2027) and Isatuximab (Cat. No. A2039) were purchased from Selleck Chemicals (Houston, TX, United States).

Flow cytometry analysis: MM cells were washed with room-temperature Phosphate-buffered saline (PBS), incubated with specific antibodies for 20 min, washed with PBS again, and acquired on Attune NxT Flow cytometry machine. The following antibodies were used for staining: CD56-APC (BD Biosciences, San Jose, CA, United States, Cat. No. 555518, Clone B159, RRID:AB_398601), CD56-PE (Miltenyi Biotec North America, Gaithersburg, MD, United States, Cat. No. 170-081-014, Clone REA196), CD38-PE (BD Biosciences, Cat. No. 555460, Clone HIT2, RRID:AB_395853), BTLA-FITC (BioLegend, Cat. No. 344524, Clone MIH26, RRID:AB_2716232), and CD274/PDL1-APC (BioLegend, Cat. No. 329708, Clone 29E.2A3, RRID:AB_940360). For surface and intracellular staining, fixation and permeabilization of MM cells was performed using Cyto-Fast™ Fix/Perm Buffer Set (BioLegend, San Diego, CA, United States). CD56 surface expression was detected with the CD56-PE antibody, while the intracellular expression was detected with the CD56-APC antibody.

Western Blot Analysis: MM cells were harvested and lysed using RIPA lysis buffer (Cell signaling, Danvers, MA, United States, Cat. No. 9806), with addition of 1mM PMSF (Cell signaling, Cat. No. 8553). Cell lysates were subjected to SDS-PAGE, transferred to nitrocellulose membranes, and immunoblotted with antibodies against the following proteins: CD56 (Cell Signaling Technology, Cat. No. 3576, RRID:AB_2149540), BCL2 (Cell Signaling Technology, Cat. No. 15071, RRID:AB_2744528), MCL1 (Cell Signaling Technology, Cat. No. 5453, RRID:AB_10694494), and GAPDH (Cell Signaling Technology, Cat. No. 3683, RRID:AB_1642205). All antibodies were diluted to a concentration of 1:1,000, except for GAPDH antibody (1: 5,000 dilution). All antibodies were prepared in milk 5% diluted in TBS-T (Biorad, Hercules, CA, United States, Cat. No. 1706435).

RNA extraction and quantitative real-time PCR analysis: RNA was extracted using TRIzol procedure (Invitrogen, Life Technologies, Carlsbad, CA, United States). After quantification, 1,000-2,000 ng of RNA were used to synthesize cDNA by ProtoScript® II First Strand cDNA Synthesis Kit (New England Biolabs, Ipswich, MA, United States) according to the manufacturer’s instructions. To evaluate the expression levels of genes of interest (see table below), quantitative real-time PCR analysis was performed using SYBR GREEN PCR Master Mix (Applied Biosystems, CA, United States) after optimization of the primer conditions. Quantitative real-time PCR analysis was performed on a ViiA 7 Real-Time PCR System (Applied Biosystems, CA, United States). Data were analyzed using the $\Delta\Delta$ Ct method. GAPDH was used for normalization.

To determine mRNA levels, the following primers were used:

**Primer name**	**Primer sequence**
GAPDH_F	GAAGGTGAAGGTCGGAGTCA

GAPDH_R	GGGGTCATTGATGGCAACAATA
BCL2_F	GGTGGGGTCATGTGTGTGG
BCL2_R	CGGTTCAGGTACTCAGTCATCC
MCL1_F isoform1	GTGCCTTTGTGGCTAAACACT
MCL1_R isoform1	AGTCCCGTTTTGTCCTTACGA
CD56 pan_F	GGCATTTACAAGTGTGTGGTTAC
CD56 pan_R	TTGGCGCATTCTTGAACATGA
CREB1_F	ATTCACAGGAGTCAGTGGATAGT
CREB1_R	CACCGTTACAGTGGTGATGG
CD38_F	GGCCCATCAGTTCACACAGG
CD38_R	GGTCATCAGCAAGGTAGCCTA
CD274_F	TGGCATTTGCTGAACGCATTT
CD274_R	TGCAGCCAGGTCTAATTGTTTT
BTLA_F	CATCTTAGCAGGAGATCCCTTTG
BTLA_R	GACCCATTGTCATTAGGAAGCA

Transient transfection of MM cell lines: U266 cells were transiently transfected using Nucleofector 4D Unit X, Kit SF, program DY-100 (Lonza). MM.1S cells were transiently transfected using Nucleofector 4D Unit X, Kit SF, program DS-137 (Lonza). After transfection, MM cells were subjected to mRNA analysis, western blotting, and other assays. The following plasmids were used: CD56 cDNA (MHS6278-202802080; clone ID number 5590188) obtained from Dharmacon, a Horizon Discovery group company (Lafayette, CO, United States); CREB1 wild-type plasmid (PT3457-5, Cat. No. 631925) obtained from Clontech Laboratories, Inc, a Takara Bio Company (Kusatsu, Shiga, Japan); and pLKO.1-TRC control was used as control

vector, called CNT in the manuscript (Addgene plasmid Cat. No. 10879, RRID: Addgene_10879).

Antibody-dependent cell-mediated cytotoxicity (ADCC) assay

MM.1S cells were transfected with either control vector (pLKO.1-TRC) or CD56 plasmid and treated with DMSO or Dara 1 μg/mL. After 24 hours, MM.1S cells were co-cultured with effector cells (peripheral blood mononuclear cells-PBMCs from n = 4 healthy donors) for 4 hours at an effector to target (E:T) ratio of 25:1 in V-shaped 96-well plates. As controls, MM.1S cells in the same conditions without the effector cells were also plated. Cells in the different conditions were then collected and stained for flow cytometry assessment, using the following antibodies: CD16-FITC (BD Biosciences, Cat. No. 555406, Clone 3G8, RRID:AB_395806), CD38-PE, CD56-APC, and LIVE/DEAD™ Fixable Aqua dye (Invitrogen, Cat. No. Catalog number: L34957). The percentage of living cells was calculated by subtracting the percentage of dead cells from 100. The percentage of Daratumumab-mediated ADCC was calculated using the following formula: % lysis = (baseline cells-treated cells)/baseline cells*100.

CD56 and CD38 clone size evaluation: The assessment of the percentage of CD56- and CD38 expressing clonal MM cells (clone size) is routinely performed for clinical purposes on each bone marrow aspirate from MM patients in the Flow Cytometry Laboratory at The Ohio State University Wexner Medical Center, which is regulated under Clinical Laboratory Improvement Amendments. Specifically, flow cytometric analysis is performed using a ten-color technique with a gating strategy based on CD45 staining and light side scatter characteristics. Plasma cells are CD138 positive, kappa/lambda restricted. A cutoff of <10% or >10% of CD56-expressing was used to define Low or High CD56 subgrouping, while the median CD38 clone size value was used as cutoff to define Low or High CD38 subgrouping.

Patient characteristics: This study was approved by the Ohio State University Institutional Review Board (OSU-23237) after providing written informed consent for the Ohio State University MM registry (OSU-10115) in accordance with the Declaration of Helsinki. Patients with a diagnosis of MM accordingly to the International Myeloma Working Group consensus criteria, who were treated at the Ohio State University Wexner Medical Center Comprehensive between January 1st, 2016 and July 1st, 2023 with a regimen containing Daratumumab-Dara (n = 152) or Isatuximab-Isa (n = 32) either as single agents or in combination with immunomodulatory drugs (IMiDs, lenalidomide or pomalidomide) or proteasome inhibitors (PIs, bortezomib and carfilzomib) were included in the retrospective analysis. There were no exclusions based on age except for individuals younger than 18 years, since the Ohio State University Comprehensive Cancer Center Multiple Myeloma clinic is an adult oncology clinic. There were no exclusions based on gender, racial, or ethnic groups for the proposed research. 1q+ status was defined based on 1q21 copy numbers present in at least 20% of the $CD138^+$ enriched cells by FISH as: 1q+ negative (2 copies), gain(1q) (3 copies), and amplification(1q) (4 copies or more). CD138 enrichment was performed using the EasySep Human CD138 Positive Selection Cocktail (STEMCELL Technologies, Vancouver, Canada).

Definition of outcomes and statistical analysis: Response criteria were based on the International Myeloma Working Group synopsis and included complete responses (CR), very good partial responses (VGPR), partial responses (PR), minimal responses (MR), stable disease (SD), or progressive disease (PD). Demographic and disease characteristics were summarized using medians and ranges for continuous variables, and frequencies and percentages for categorical variables and compared using chi-square test or Fisher’s exact test. Primary endpoints were progression-free survival (PFS) from first day of Dara or Isa-based regimens to progression or death censoring the patients without progression at last follow-up. Overall survival

(OS) was calculated from the first day of therapy with Dara or Isa at last follow-up or death. PFS was estimated using the Kaplan-Meier method, using Log-rank test to compare the groups. Cox proportional hazard regression models were used to estimate the hazard ratios for risk of progression or death. The multivariable Cox model was built including all the variables significantly associated with PFS in the univariable analysis or variables known to affect outcomes in MM. Analyses were performed using IBM SPSS Statistics version 28, and all statistical tests were two-sided with statistical significance at 0.05.

RNA-sequencing Analysis: RNA-sequencing data were analyzed from the MMRF CoMMpass database. The MMRF data were generated as part of the Multiple Myeloma Research Foundation Personalized Medicine Initiatives. CD56 (ENSG00000149294) and CD38 (ENSG00000004468) FKPM levels were normalized to Log_2 values and correlated as dependent variables in the linear regression analysis, or with 1q21+ status. For Gene set enrichment analysis (GSEA), patients were divided based on median CD56 levels. GSEA was used to score enrichment levels and significance of a predefined immune signature reported in **Table S4** and derived from Chen et al (Ref. 8). Volcano plot analysis was used to show differences in expression and adjusted *p*-values.

Supplementary Table S1. Characteristics of n = 152 patients treated with Daratumumab.

	All patients (n = 152)	<10% CD56-expressing clonal MM cells (n = 48)	>10% CD56-expressing clonal MM cells (n = 104)	*p* values
**CD56 clone size,** Median (range)	42.9 (0-99.8)	1.0 (0-10)	75 (11.9-99.8)	<.001
**CD38 clone size,** Median (range)	97.25 (0-100)	86.25 (0-100)	98.45 (0-100)	0.126
**Age,** Median (range)	66 (38-87)	66 (38-83)	66.5 (38-85)	0.34
**Gender,** no (%)				
Male	98 (64.5)	31 (64.6)	67 (64.4)	0.985
Female	54 (35.5)	17 (35.4)	37 (35.6)	
**Race,** no (%)				
NHW	126 (82.9)	35 (72.9)	91 (87.5)	0.07
NHB	21 (13.8)	10 (20.8)	11 (10.6)	
Other	5 (3.3)	3 (6.3)	2 (1.9)	
**MM type,** no (%)				
IgG	84 (55.3)	31 (64.6)	53 (51)	0.251
IgA	37 (24.3)	11 (22.9)	26 (25)	
LC	27 (17.8)	6 (12.5)	21 (20.2)	
Other	4 (2.6)	0	4 (3.8)	
**ISS,** no (%)				
I	76 (50)	23 (47.9)	53 (51)	0.140
II	46 (30.3)	19 (39.6)	27 (26)	
III	30 (19.7)	6 (12.5)	24 (23)	
**Cytogenetic profile,** no (%)				
Standard Risk	127 (83.6)	40 (83.3)	87 (83.6)	0.960
High risk	25 (16.4)	8 (16.7)	17 (16.4)	
**t(11;14),** no (%)				
Yes	23 (15.2)	11 (22.9)	12 (11.5)	0.069
No	129 (84.8)	37 (77.1)	92 (88.5)	
**t(4;14),** no (%)				
Yes	11 (7.3)	1 (2.1)	10 (9.6)	0.096
No	141 (92.7)	47 (97.9)	94 (90.4)	
**del(13q),** no (%)				
Yes	70 (46)	24 (50)	46 (44.2)	0.507
No	82 (54)	24 (50)	58 (55.8)	
**del(17p),** no (%)				
Yes	11 (7.9)	3 (6.3)	8 (8.7)	0.738
No	140 (92.1)	45 (93.7)	95 (91.3)	
**1q+,** no (%)				
Yes	50 (32.9)	15 (31.3)	35 (33.7)	0.769
No	102 (67.1)	33 (68.7)	69 (66.3)	
**Prior lines,** Median (range)	3 (2-19)	4 (2-19)	3 (2-14)	0.074
**Combination agent,** no (%)				
IMiDs	84 (55.7)	24 (50)	60 (57.7)	0.477
PIs	38 (24.1)	15 (31.2)	23 (22.1)	
Single	30 (20.2)	9 (18.8)	21 (20.2)	
**Best response,** no (%)				
CR/VGPR	67 (44.1)	14 (30)	53 (51)	0.012
PR/MR	85 (55.9)	34 (70)	51 (49)	
**Progression,** no (%)				
Yes	95 (62.5)	36 (75)	59 (56.7)	0.03
No	57 (37.5)	12 (25)	45 (43.3)	

Abbreviations: n, number; MM, Multiple Myeloma; *p*, p-value; NHW, non-Hispanic white; NHB, non-Hispanic black; LC, light chain disease; ISS, International staging system; IMiDs, immunomodulatory drugs; PIs, proteasome inhibitors; CR, complete response; VGPR, very good partial response; PR, partial response; MR, minimal response.

Supplementary Table S2. Characteristics of n = 84 patients treated with Daratumumab in combination with immunomodulatory drugs (IMiDs).

	All patients (n = 84)	<10% CD56-expressing clonal cells (n = 24)	>10% CD56-expressing clonal cells (n = 60)	*p* values
**CD56 clone size,** Median (range)	57.5 (0-99.6)	1 (0-10)	76.3 (11.9-99.60)	<0.001
**Age,** Median (range)	67 (38-84)	67.5 (53-83)	66.5 (38-84)	0.224
**Gender,** no (%)				
Male	56 (66.7)	16 (66.7)	40 (66.7)	1.00
Female	28 (33.3)	8 (33.3)	20 (33.3)	
**Race,** no (%)				
NHW	74 (88.1)	20 (83.3)	54 (90)	0.261
NHB	9 (10.7)	3 (12.5)	6 (10)	
Other	1 (1.2)	1 (4.2)	0	
**MM type,** no (%)				
IgG	43 (51.2)	12 (50)	31 (51.7)	0.901
IgA	25 (29.8)	7 (29.2)	18 (30)	
LC	15 (17.8)	5 (20.8)	10 (16.7)	
Other	1 (1.2)	0	1 (1.6)	
**ISS,** no (%)				
I	44 (52.4)	12 (50)	32 (53.3)	0.106
II	20 (23.8)	9 (37.5)	11 (18.3)	
III	20 (23.8)	3 (12.5)	17 (28.4)	
**Cytogenetic profile,** no (%)				
Standard Risk	72 (85.7)	21 (87.5)	51 (85)	0.767
High risk	12 (14.3)	3 (12.5)	9 (15)	
**t(11;14),** no (%)				
Yes	13 (15.5)	7 (29.2)	6 (10)	0.028
No	71 (84.5)	17 (70.8)	54 (90)	
**t(4;14),** no (%)				
Yes	5 (5.9)	0 (0)	5 (8.3)	0.145
No	79 (94.1)	24 (100)	55 (91.7)	
**del(13q),** no (%)				
Yes	36 (42.8)	10 (41.7)	26 (43.3)	0.889
No	48 (57.2)	14 (58.3)	34 (56.7)	
**del(17p),** no (%)				
Yes	7 (8.3)	1 (4.2)	6 (10)	0.372
No	77 (91.7)	23 (95.8)	54 (90)	
**1q+,** no (%)				
Yes	27 (32.1)	7 (29.2)	20 (33.3)	0.712
No	57 (67.9)	17 (70.8)	40 (66.7)	
**Prior lines,** Median (range)	3 (2-14)	3.5 (2-9)	3.0 (2-14)	0.718
**Best response,** no (%)				
CR/VGPR	39 (46.4)	9	30 (50)	0.23
PR/MR	45 (53.6)	15	30 (50)	
**Progression,** no (%)				
Yes	51 (31.6)	17 (70.8)	34 (56.7)	0.23
No	33 (68.4)	7 (29.2)	26 (43.3)	

Abbreviations: n, number; MM, Multiple Myeloma; *p*, p-value; NHW, non-Hispanic white; NHB, non-Hispanic black; LC, Light chain disease; ISS, International staging system; IMiD, immunomodulatory drugs; CR, complete response; VGPR, very good partial response; PR, partial response; MR, minimal response.

Supplementary Table S3. Median PFS based on CD38, and CD56 clone size subgrouping.

All patients (n = 152)	Median	95% CI
**Low CD38, Low CD56 (n = 30)**	24.00	5.5-42.5
**High CD38, Low CD56 (n = 18)**	8.7	3.7-13.8
**Low CD38, High CD56 (n = 46)**	45.2	19.0-71.3
**High CD38, High CD56 (n = 58)**	21.2	11.5-30.8

Dara-IMiD treated patients (n = 84)	Median	95% CI
**Low CD38, Low CD56 (n = 14)**	32.5	12.9-52.1
**High CD38, Low CD56 (n = 10)**	8.7	0.6-16.8
**Low CD38, High CD56 (n = 25)**	21.3	4-50.8
**High CD38, High CD56 (n = 35)**	21.1	11.9-30.5

Abbreviations: PFS, progression-free survival; n, number; CI, confidence interval; IMiD, immunomodulatory drugs.

Supplementary Table S4. Immune signature genes related to **Fig. 2D** and **Fig. S5A**.

Gene name	NCBI ID	Probe gene name
B2M	567	ENSG00000166710
BTLA	151888	ENSG00000186265
CCL17	6361	ENSG00000102970
CCL18	6362	ENSG00000275385
CCL19	6363	ENSG00000172724
CCL2	6347	ENSG00000108691
CCL21	6366	ENSG00000137077
CCL22	6367	ENSG00000102962
CCL3	6348	ENSG00000277632
CCL4	6351	ENSG00000275302
CCL5	6352	ENSG00000271503
CCL8	6355	ENSG00000108700
ITGAM (CD11B)	3684	ENSG00000169896
ITGAX (CD11C)	3687	ENSG00000140678
FUT4 (CD15)	2526	ENSG00000196371
FCGR3A (CD16)	2214	ENSG00000162747
CD1D	912	ENSG00000158473
IL2RA (CD25)	3559	ENSG00000172183
CD27	939	ENSG00000139193
CD274 (PDL1)	29126	ENSG00000120217
CD276	80381	ENSG00000103855

CD28	940	ENSG00000178562
CD33	945	ENSG00000105383
CD3G	917	ENSG00000160654
CD4	920	ENSG00000010610
CD47	961	ENSG00000196776
CD40	958	ENSG00000101017
CD40LG	959	ENSG00000102245
NCAM1 (CD56)	4684	ENSG00000149294
CD58	965	ENSG00000116815
CD70	970	ENSG00000125726
CD8A	925	ENSG00000153563
CD80	941	ENSG00000121594
CD86	942	ENSG00000114013
CD8B	926	ENSG00000172116
CTLA4	1493	ENSG00000163599
CXCL11	6373	ENSG00000169248
CXCL13	10563	ENSG00000156234
LAMP3	27074	ENSG00000078081
FAS	355	ENSG00000026103
FASLG	356	ENSG00000117560
FOXP3	50943	ENSG00000049768
CSF3 (GCSF)	1440	ENSG00000108342
CSF2 (GMCSF)	1437	ENSG00000164400
GZMB	3002	ENSG00000100453
HAVCR2 (TIM3)	84868	ENSG00000135077
HLA-A	3105	ENSG00000206503
HLA-B	3106	ENSG00000234745
HLA-C	3107	ENSG00000204525
HLA-DPB1	3115	ENSG00000223865
HLA-DQA1	3117	ENSG00000196735
HLA-DRB1	3123	ENSG00000196126
HLA-E	3133	ENSG00000204592
ICOS	29851	ENSG00000163600
IDO1	3620	ENSG00000131203

IFNA1	3439	ENSG00000197919
IL10	3586	ENSG00000136634
IL13	3596	ENSG00000169194
IL17A	3605	ENSG00000112115
IL2	3558	ENSG00000109471
IL22	50616	ENSG00000127318
IL2RA	3559	ENSG00000134460
IL5	3567	ENSG00000113525
IL6	3569	ENSG00000136244
IL7R	3575	ENSG00000168685
KIR2DL1	3802	ENSG00000125498
LAG3	3902	ENSG00000089692
CSF1 (MCSF)	1435	ENSG00000184371
MICA	4276	ENSG00000204520
MICB	4277	ENSG00000204516
PDCD1	5133	ENSG00000188389
PDCD1LG2	80380	ENSG00000197646
TNF	7124	ENSG00000232810
TNFRSF14	8764	ENSG00000157873
TNFRSF4 (OX40)	7293	ENSG00000186827
TNFRSF9	3604	ENSG00000049249
TNFSF4 (OX40L)	7292	ENSG00000117586
TNFSF9	8744	ENSG00000125657
VTCN1	79679	ENSG00000134258
IL12A (NKSF1)	3592	ENSG00000168811
HGF	3082	ENSG00000019991
IL16	3603	ENSG00000172349
CXCL10	3627	ENSG00000169245
CXCL12	6387	ENSG00000107562
IL3	3562	ENSG00000164399
IFNG	3458	ENSG00000111537
TNFSF10 (TRAIL)	8743	ENSG00000121858
PGF	5228	ENSG00000119630

Supplementary Table S5. Characteristics of n = 32 patients treated with Isatuximab.

	**All patients (n = 32)**	**<10% CD56-expressing clonal MM cells (n = 13)**	**>10% CD56-expressing clonal MM cells (n = 19)**	***p* values**
**CD56 clone size,** Median (range)	42.55 (0-99.6)	1 (0-5.2)	83.6 (17-99.6)	< 0.0001
**Age,** Median (range)	69 (46-83)	71 (57-83)	66 (46-75)	0.503
**Gender,** no (%)				
Male	17 (53.1)	5 (38.5)	12 (63.2)	0.169
Female	15 (46.9)	8 (61.5)	7 (36.8)	
**Race,** no (%)				
NHW	27 (84.3)	10 (76.9)	17 (89.5)	0.337
NHB	5 (15.7)	3 (23.1)	2 (10.5)	
**MM type,** no (%)				
IgG	12 (37.5)	4 (30.8)	8 (42.1)	0.454
IgA	11 (34.4)	4 (30.8)	7 (36.8)	
LC	8 (25)	5 (38.4)	3 (15.8)	
Other	1 (3.1)	0	1 (5.3)	
**ISS,** no (%)				
I	14 (43.7)	6 (46.1)	8 (42.1)	0.027
II	11 (34.4)	7 (53.9)	4 (21.0)	
III	7 (21.9)	0	7 (36.9)	
**Cytogenetic profile**				
Standard Risk	25 (78.1)	12 (92.3)	13 (68.4)	0.108
High risk	7 (21.9)	1 (7.7)	6 (31.6)	
**t(11;14),** no (%)				
Yes	6 (18.7)	5 (38.5)	1 (5.3)	0.018
No	26 (81.3)	8 (61.5)	18 (94.7)	
**t(4;14),** no (%)				
Yes	4 (12.5)	0	4 (21)	0.077
No	28 (87.5)	13 (100)	15 (79)	
**del(13q),** no (%)				
Yes	16 (50)	6 (46.1)	10 (52.6)	0.719
No	16 (50)	7 (53.9)	9 (47.4)	
**del(17p),** no (%)				
Yes	6 (18.7)	1 (7.7)	5 (26.3)	0.185
No	26 (81.3)	12 (92.3)	14 (73.7)	
**1q+,** no (%)				
Yes	13 (40.6)	6 (46.1)	13 (68.4)	0.208
No	19 (59.4)	7 (53.9)	6 (31.6)	
**Prior lines,** Median (range)	8 (3-19)	9 (3-19)	7 (3-19)	0.460
**Combination agent,** no (%)				
pomalidomide	24 (75)	8 (61.5)	16 (84.2)	0.148
carfilzomib	8 (25)	5 (38.5)	3 (15.8)	
**Progression,** no (%)				
Yes	24 (75)	9 (69.2)	15 (78.9)	0.533
No	8 (25)	4 (30.8)	4 (21.1)	

Abbreviations: n, number; MM, Multiple Myeloma; *p*, p-value; NHW, non-Hispanic white; NHB, non-Hispanic black; LC, light chain disease; ISS, International staging system.

Supplementary Table S6. Median PFS based on CD56 clone size, and 1q+ status subgrouping.

All patients (n = 152)	Median	95% CI
**Low CD56, 1q+ neg (n = 33)**	24.00	14.3-33.7
**Low CD56, 1q+ (n = 15)**	6.7	3.2-10.3
**High CD56, 1q+ neg (n = 69)**	31.8	11.8-26.5
**High CD56, 1q+ (n = 35)**	19.2	14.8-27.5

Dara-IMiD treated patients (n = 84)	Median	95% CI
**Low CD56, 1q+ neg (n = 17)**	22.3	0-45.7
**Low CD56, 1q+ (n = 7)**	3.9	0.4-7.4
**High CD56, 1q+ neg (n = 40)**	36.8	16.7-56.8
**High CD56, 1q+ (n = 20)**	15.3	10.5-20.2

Abbreviations: PFS, progression-free survival; n, number; CI, confidence interval; IMiD, immunomodulatory drugs.

3. Supplementary Figures:

Supplementary Figure S1. Modulation of CD56 and its downstream targets by anti-CD38 monoclonal antibodies. A. Surface expression of CD56 in OPM-2 and H929 cells treated with DMSO or Isa 1 μg/mL for 48 hours. Mean fluorescence intensity (MFI) value ratios are obtained normalizing to DMSO controls. n = 3 replicates. OPM-2 $p = 0.05$ (*); H929 $p = 0.0023$ (**). **B.** OPM-2 cells treated with increasing doses of Dara for 48 hours. One representative experiment is shown. **C.** Surface staining of CD56 after 1 hour of treatment with DMSO or Dara 1 μg/mL. MFI value ratios are obtained normalizing to DMSO controls. n = 3 replicates. OPM-2 $p = 0.0032$ (**); H929 $p < 0.0001$ (****). **D.** Representative plots of surface and intracellular staining in OPM-2 cells treated with DMSO or Dara 1 μg/mL for 1 hour. **E.** Intracellular staining of CD56 in OPM-2 and H929 cells treated with DMSO or Isa 1 μg/mL for 1 hour. MFI value ratios are obtained normalizing to DMSO controls. n = 3 replicates. *p* values are not significant (ns). **F.** Western blot analysis for MCL1, BCL2, and GAPDH in OPM-2 cells and H929 cells treated with DMSO and Isa 1 μg/mL for 48 hours. **G.** Western blot analysis for CD56, MCL1, and GAPDH in U266 control cells (CNT) and U266 cells overexpressing CD56 treated with DMSO or Dara 1 μg/mL for 48 hours. Optical density (OD) ratio normalized to CNT + DMSO is reported in the figure. **H.** Fold changes of MCL1 mRNA levels in U266 control cells (CNT + DMSO - gray) or U266 cells overexpressing CD56 treated with DMSO (CD56 + DMSO - light blue) or Dara 1 μg/mL (CD56 + Dara - dark blue) for 48 hours. n = 2 replicates. CD56 versus CD56 + Dara $p = 0.0160$ (*).

Supplementary Figure S2. Outcomes of patients treated with Dara based on CD56 clone size. A. Clone sizes of CD38- and CD56-expressing MM cells in n = 152 patients. Median CD38 clone size value = 97.25% (black line); median CD56 clone size value = 42.90% (black line). 25th and 75th percentiles are shown in red. **B.** Overall survival (OS) from the first day of Dara therapy until death or last follow-up in patients with less (n = 48, Low CD56-blue) or more than

10% of CD56-expressing MM clonal cells (n = 104, High CD56-fuchsia). Median OS and 95% confidence interval (CI) are reported in the insert of the plot. Log-rank $p = 0.322$. **C.** PFS from the first day of Dara-proteasome inhibitor (PI) therapy in patients with less (n = 15, Low CD56-blue) or more than 10% of CD56-expressing MM clonal cells (n = 23, High CD56-fuchsia). Median PFS and 95% CI are reported in the insert of the plot. Log-rank $p = 0.690$. **D.** PFS from the first day of Dara single agent therapy in patients with less (n = 9, Low CD56-blue) or more than 10% of CD56-expressing MM clonal cells (n = 21, High CD56-fuchsia). Median PFS and 95% CI are reported in the insert of the plot. Log-rank $p = 0.334$.

Supplementary Figure S3. Role of CD38 and CD56 correlation in the response to Dara. A. Progression-free survival (PFS) from the first day of Dara-IMiD therapy in patients with Low CD38, Low CD56 (n = 14, blue), High CD38, Low CD56 (n = 10, black), Low CD38, High CD56 (n = 25, fuchsia), and High CD38, High CD56 (n = 35, orange). Median PFS and 95% confidence interval (CI) are reported in **Table S3**. Log-rank $p = 0.002$. **B.** Log_2 CD38 expression values in patients with Low or High CD56 Log_2 expression in the CoMMpass MMRF database. Total patients = 809, $p < 0.0001$ (****). Blue lines indicate median values. Dotted black lines indicate the 25th and 75th percentiles. **C.** Fold changes of CD56 mRNA levels in U266 control cells (CNT) or U266 cells overexpressing CD56 in the 4 replicates used in the manuscript. Ratio is normalized to the control cells. $p = 0.0080$ (**). **D.** Flow cytometry staining for CD56 to confirm overexpression of CD56 in U266 cells. **E.** Fold changes of CD56 mRNA levels in MM.1S control cells (CNT) or MM.1S cells overexpressing CD56 in the 2 replicates used in the manuscript. Ratio is normalized to the control cells. $p = 0.038$ (*). **F.** Fold changes of CD38 Mean Fluorescence Intensity (MFI) and mRNA levels in MM.1S control cells (CNT) or MM.1S cells overexpressing CD56. Ratio is normalized to the control cells. n = 2 replicates. MFI $p = 0.04$ (*); mRNA $p = 0.028$ (*).

Supplementary Figure S4. CREB1 does not regulate CD38 expression in MM.

A. Fold changes of CREB1 mRNA levels in U266 control cells (CNT) or U266 cells overexpressing CREB1. Ratio is normalized to the control cells. n = 3 replicates. $p = 0.006$ (**). **B.** Fold changes of CD38 MFI and mRNA levels in U266 control cells (CNT) or U266 cells overexpressing CREB1. Ratio is normalized to the control cells. n = 2, 3 replicates. MFI $p = 0.0058$ (**); mRNA $p = 0.0007$ (***).

Supplementary Figure S5. CD56 regulates immune markers in MM. A. Pathway analysis of the “Immune signature” from Chen et al (ref. 8) in the MMRF CoMMpass dataset. Patients are divided based on median cutoff of CD56 expression. NES, normalized enrichment score and *p* value are reported in the figure. **B.** Fold changes of BTLA and PDL1 Mean Fluorescence Intensity (MFI) values in U266 control cells (CNT) or U266 cells overexpressing CD56. Ratio is normalized to the control cells. n = 2 replicates. BTLA $p = 0.0016$ (**); PDL1 $p = 0.034$ (*). **C.** Fold changes of BTLA and PDL1 MFI values in MM.1S control cells (CNT) or MM.1S cells overexpressing CD56. Ratio is normalized to the control cells. n = 2 replicates. BTLA $p = 0.0004$ (***); PDL1 $p = 0.0097$ (**).

Supplementary Figure S6. Role of CD56 and 1q+ status in the response to anti-CD38 monoclonal antibodies. A. Progression-free survival (PFS) from the first day of Isa therapy in patients with less (n = 13, Low CD56-blue) or more than 10% of CD56-expressing MM clonal cells (n = 19, High CD56-fuchsia). Median PFS and 95% CI are reported in the insert of the plot. Log-rank $p = 0.31$. **B.** PFS from the first day of Isa therapy in patients 1q+ neg (n = 13, yellow), patients with gain(1q) (n = 12, crimson), or amp(1q) (n = 7, orange). Median PFS and 95% Confidence interval (CI) are reported in the insert. Log-rank $p = 0.06$. **C.** PFS from the first day of Dara therapy in patients 1q+ neg (n = 102, yellow), patients with gain(1q) (n = 34, crimson), or amp(1q) (n = 16, orange). Median PFS and 95% CI are reported in the insert. Log-rank *p* <

0.001. **D.** Clone sizes of CD38- and CD56-expressing MM cells in 77 patients with 1q+ (either gains or amplifications) or 156 patients with normal 1q copy number (1q+ neg) in our MM database. CD38 1q+ versus 1q+ neg: $p = 0.01$ (*); CD56 1q+ versus 1q+ neg: $p = 0.38$, ns. Black solid lines represent median values. Black dotted lines represent the 25th and 75th percentiles. **E.** PFS from the first day of Dara-IMiD therapy in patients with Low CD56, 1q+ neg (n = 17, blue), Low CD56, 1q+ (n = 7, dark green), High CD56, 1q+ neg (n = 40, purple), and High CD56, 1q+ (n = 20, orange) disease. Median PFS and 95% CI are reported in **Table S6**. Log-rank p = 0.003.

Fig. S1

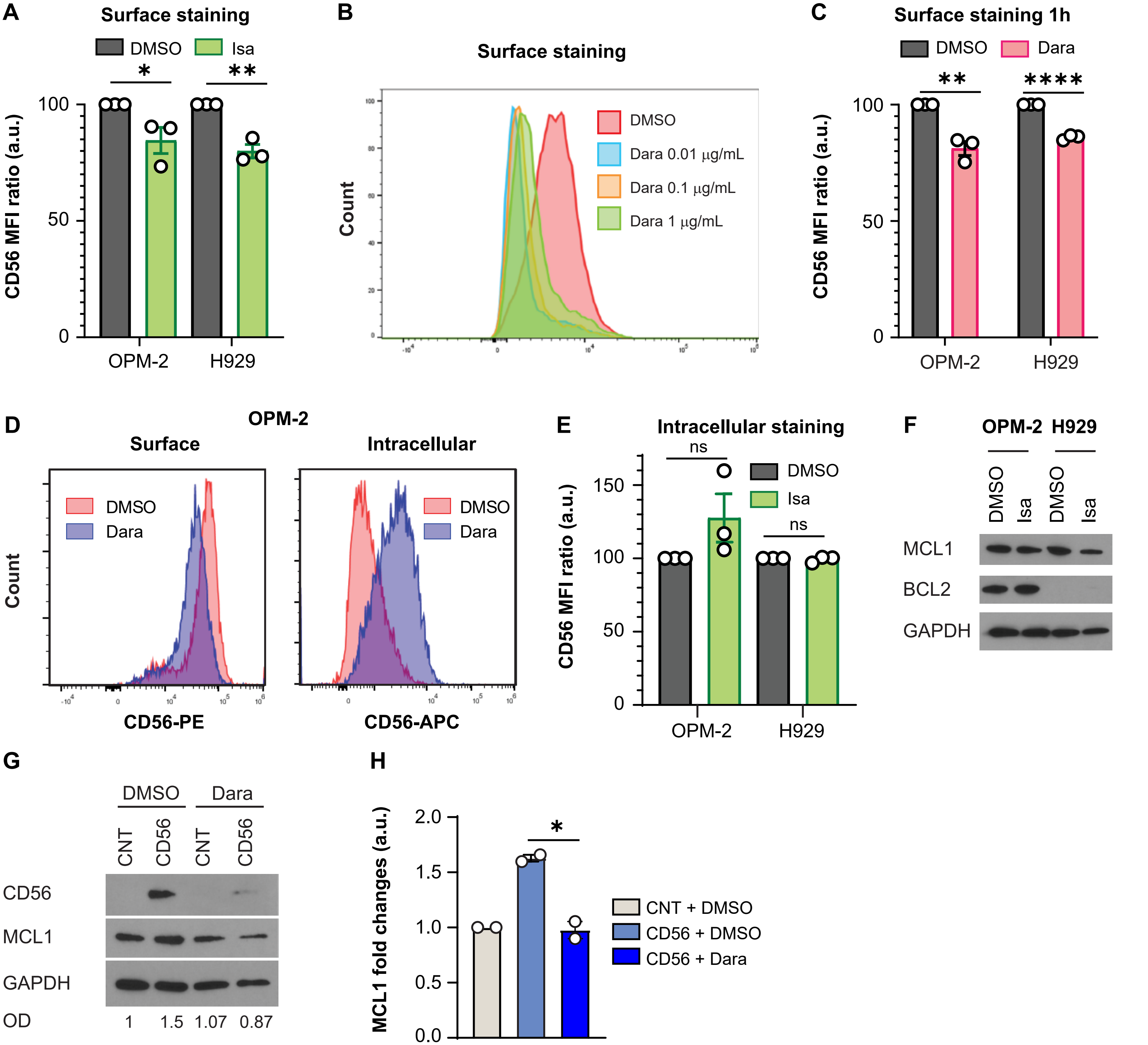

Supplementary Figure S1. Modulation of CD56 and its downstream targets by anti-CD38 monoclonal antibodies.

A. Surface expression of CD56 in OPM-2 and H929 cells treated with DMSO or Isa 1 μg/mL for 48 hours. Mean fluorescence intensity (MFI) value ratios are obtained normalizing to DMSO controls. n = 3 replicates. OPM-2 p = 0.05 (*); H929 p = 0.0023 (**).
B. OPM-2 cells treated with increasing doses of Dara for 48 hours. One representative experiment is shown.
C. Surface staining of CD56 after 1 hour of treatment with DMSO or Dara 1 μg/mL. MFI value ratios are obtained normalizing to DMSO controls. n = 3 replicates. OPM-2 p = 0.0032 (**); H929 p < 0.0001 (****).
D. Representative plots of surface and intracellular staining in OPM-2 cells treated with DMSO or Dara 1 μg/mL for 1 hour.
E. Intracellular staining of CD56 in OPM-2 and H929 cells treated with DMSO or Isa 1 μg/mL for 1 hour. MFI value ratios are obtained normalizing to DMSO controls. n = 3 replicates. p values are not significant (ns).
F. Western blot analysis for MCL1, BCL2, and GAPDH in OPM-2 cells and H929 cells treated with DMSO and Isa 1 μg/mL for 48 hours.
G. Western blot analysis for CD56, MCL1, and GAPDH in U266 control cells (CNT) and U266 cells overexpressing CD56 treated with DMSO or Dara 1 μg/mL for 48 hours. Optical density (OD) ratio normalized to CNT + DMSO is reported in the figure.
H. Fold changes of MCL1 mRNA levels in U266 control cells (CNT + DMSO - gray) or U266 cells overexpressing CD56 treated with DMSO (CD56 + DMSO - light blue) or Dara 1 μg/mL (CD56 + Dara - dark blue) for 48 hours. n = 2 replicates. CD56 versus CD56 + Dara p = 0.0160 (*).

Fig. S2

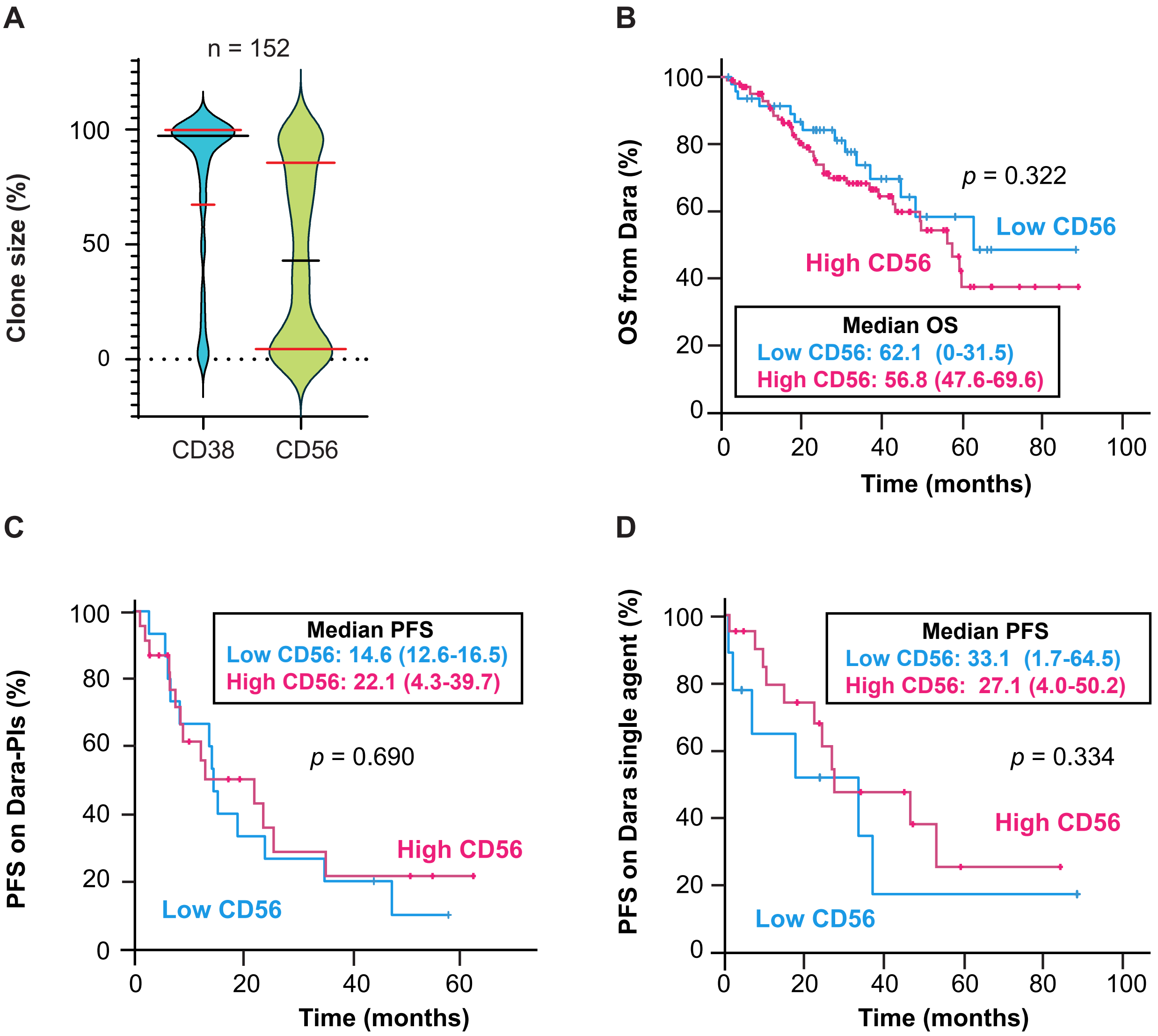

Supplementary Figure S2. Outcomes of patients treated with Dara based on CD56 clone size.

A. Clone sizes of CD38- and CD56-expressing MM cells in n = 152 patients. Median CD38 clone size value = 97.25% (black line); median CD56 clone size value = 42.90% (black line). 25th and 75th percentiles are shown in red.
B. Overall survival (OS) from the first day of Dara therapy until death or last follow-up in patients with less (n = 48, Low CD56-blue) or more than 10% of CD56-expressing MM clonal cells (n = 104, High CD56-fuchsia). Median OS and 95% confidence interval (CI) are reported in the insert of the plot. Log-rank $p = 0.322$.
C. PFS from the first day of Dara-proteasome inhibitor (PI) therapy in patients with less (n = 15, Low CD56-blue) or more than 10% of CD56-expressing MM clonal cells (n = 23, High CD56-fuchsia). Median PFS and 95% CI are reported in the insert of the plot. Log-rank $p = 0.690$.
D. PFS from the first day of Dara single agent therapy in patients with less (n = 9, Low CD56-blue) or more than 10% of CD56-expressing MM clonal cells (n = 21, High CD56-fuchsia). Median PFS and 95% CI are reported in the insert of the plot. Log-rank $p = 0.334$.

Fig. S3

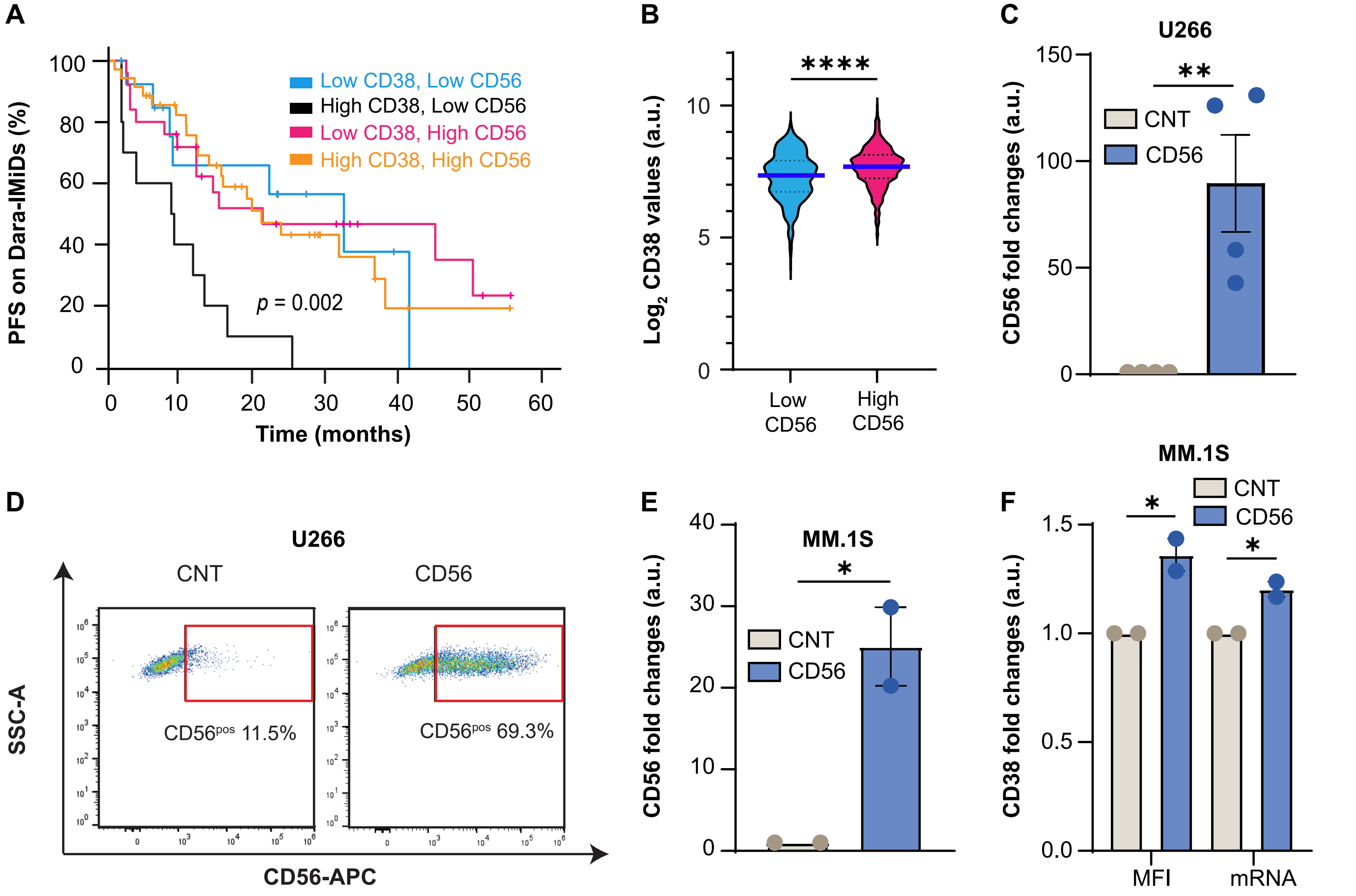

Supplementary Figure S3. Role of CD38 and CD56 correlation in the response to Dara.

A. Progression-free survival (PFS) from the first day of Dara-IMiD therapy in patients with Low CD38, Low CD56 (n = 14, blue), High CD38, Low CD56 (n = 10, black), Low CD38, High CD56 (n = 25, fuchsia), and High CD38, High CD56 (n = 35, orange). Median PFS and 95% confidence interval (CI) are reported in **Table S3**. Log-rank *p* = 0.002.
B. Log_2 CD38 expression values in patients with Low or High CD56 Log_2 expression in the CoMMpass MMRF database. Total patients = 809, p < 0.0001 (****). Blue lines indicate median values. Dotted black lines indicate the 25th and 75th percentiles.
C. Fold changes of CD56 mRNA levels in U266 control cells (CNT) or U266 cells overexpressing CD56 in the 4 replicates used in the manuscript. Ratio is normalized to the control cells. *p* = 0.0080 (**).
D. Flow cytometry staining for CD56 to confirm overexpression of CD56 in U266 cells.
E. Fold changes of CD56 mRNA levels in MM.1S control cells (CNT) or MM.1S cells overexpressing CD56 in the 2 replicates used in the manuscript. Ratio is normalized to the control cells. *p* = 0.038 (*).
F. Fold changes of CD38 Mean Fluorescence Intensity (MFI) and mRNA levels in MM.1S control cells (CNT) or MM.1S cells overexpressing CD56. Ratio is normalized to the control cells. n = 2 replicates. MFI *p* = 0.04 (*); mRNA *p* = 0.028 (*).

Fig. S4

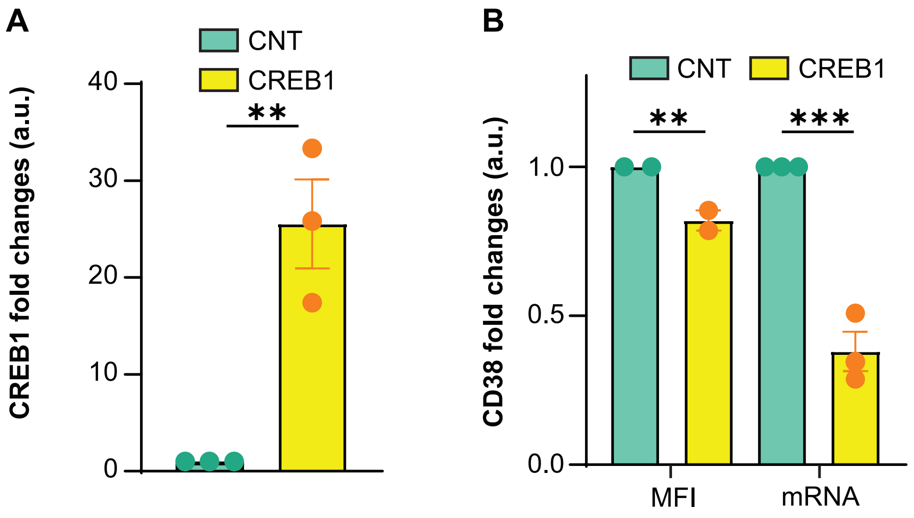

Supplementary Figure S4. CREB1 does not regulate CD38 expression in MM.

A. Fold changes of CREB1 mRNA levels in U266 control cells (CNT) or U266 cells overexpressing CREB1. n = 3 replicates. Ratio is normalized to the control cells. p = 0.006 (**).
B. Fold changes of CD38 MFI and mRNA levels in U266 control cells (CNT) or U266 cells overexpressing CREB1. Ratio is normalized to the control cells. n = 2, 3 replicates. MFI p = 0.0058 (**); mRNA p = 0.0007 (***).

Fig. S5

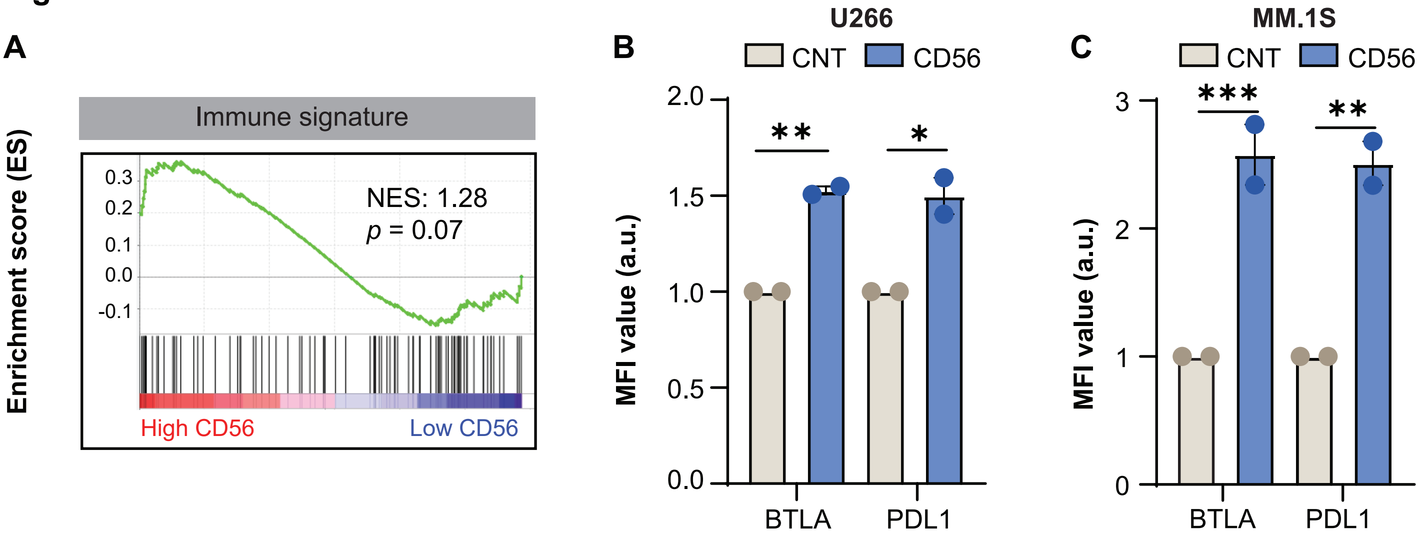

Supplementary Figure S5. CD56 regulates immune markers in MM.

A. Pathway analysis of the “Immune signature” from Chen et al (ref. 8) in the MMRF CoMMpass dataset. Patients are divided based on median cutoff of CD56 expression. NES, normalized enrichment score and *p* value are reported in the figure.
B. Fold changes of BTLA and PDL1 Mean Fluorescence Intensity (MFI) values in U266 control cells (CNT) or U266 cells overexpressing CD56. Ratio is normalized to the control cells. n = 2 replicates. BTLA *p* = 0.0016 (**); PDL1 *p* = 0.034 (*).
C. Fold changes of BTLA and PDL1 MFI values in MM.1S control cells (CNT) or MM.1S cells overexpressing CD56. Ratio is normalized to the control cells. n = 2 replicates. BTLA *p* = 0.0004 (***); PDL1 *p* = 0.0097 (**).

Fig. S6

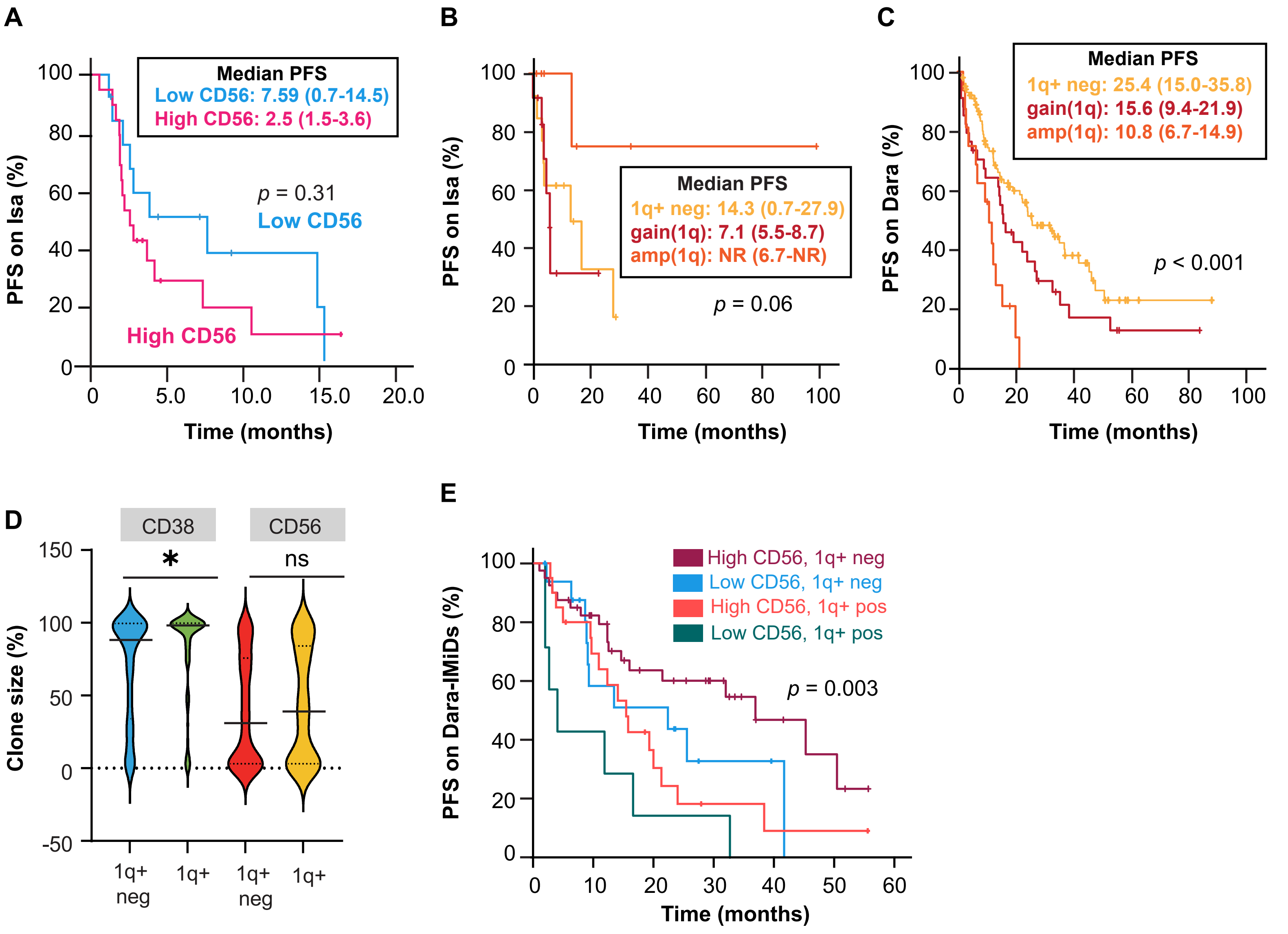

Supplementary Figure S6. Role of CD56 and 1q+ status in the response to anti-CD38 monoclonal antibodies.

A. Progression-free survival (PFS) from the first day of Isa therapy in patients with less (n = 13, Low CD56-blue) or more than 10% of CD56-expressing MM clonal cells (n = 19, High CD56-fuchsia). Median PFS and 95% CI are reported in the insert of the plot. Log-rank *p* = 0.31.
B. PFS from the first day of Isa therapy in patients 1q+ neg (n = 13, yellow), patients with gain(1q) (n = 12, crimson), or amp(1q) (n = 7, orange). Median PFS and 95% Confidence interval (CI) are reported in the insert. Log-rank *p* = 0.06.
C. PFS from the first day of Dara therapy in patients 1q+ neg (n = 102, yellow), patients with gain(1q) (n = 34, crimson), or amp(1q) (n = 16, orange). Median PFS and 95% CI are reported in the insert. Log-rank *p* < 0.001.
D. Clone sizes of CD38- and CD56-expressing MM cells in 77 patients with 1q+ (either gains or amplifications) or 156 patients with normal 1q copy number (1q+ neg) in our MM database. CD38 1q+ versus 1q+ neg: *p* = 0.01 (*); CD56 1q+ versus 1q+ neg: *p* = 0.38, ns. Black solid lines represent median values. Black dotted lines represent the 25th and 75th percentiles.
E. PFS from the first day of Dara-IMiD therapy in patients with Low CD56, 1q+ neg (n = 17, blue), Low CD56, 1q+ (n = 7, dark green), High CD56, 1q+ neg (n = 40, purple), and High CD56, 1q+ (n = 20, orange) disease. Median PFS and 95% CI are reported in **Table S6**. Log-rank *p* = 0.003.
